# Model biases in rice phenology under warmer climates

**DOI:** 10.1038/srep27355

**Published:** 2016-06-07

**Authors:** Tianyi Zhang, Tao Li, Xiaoguang Yang, Elisabeth Simelton

**Affiliations:** 1 State Key Laboratory of Atmospheric Boundary Layer Physics and Atmospheric Chemistry, Institute of Atmospheric Physics, Chinese Academy of Sciences, Beijing, 100029, China; 2 International Rice Research Institute, Los Baños, Philippines; 3 College of Resources and Environmental Sciences, China Agricultural University, Beijing, China; 4 World Agroforestry Centre (ICRAF), Ha Noi, Vietnam; 5 Sustainability Research Institute, University of Leeds, Leeds, UK

## Abstract

Climate-induced crop yields model projections are constrained by the accuracy of the phenology simulation in crop models. Here, we use phenology observations from 775 trials with 19 rice cultivars in 5 Asian countries to compare the performance of four rice phenology models (growing-degree-day (GDD), exponential, beta and bilinear models) when applied to warmer climates. For a given cultivar, the difference in growing season temperature (GST) varied between 2.2 and 8.2 °C in different trials, which allowed us to calibrate the models for lower GST and validate under higher GST, with three calibration experiments. The results show that in warmer climates the bilinear and beta phenology models resulted in gradually increasing bias for phenology predication and double yield bias per percent increase in phenology simulation bias, while the GDD and exponential models maintained a comparatively constant bias. The phenology biases were primarily attributed to varying phenological patterns to temperature in models, rather than on the size of the calibration dataset. Additionally, results suggest that model simulations based on multiple cultivars provide better predictability than using one cultivar. Therefore, to accurately capture climate change impacts on rice phenology, we recommend simulations based on multiple cultivars using the GDD and exponential phenology models.

Capturing warming effects on rice phenology is vital for obtaining reliable assessments of climate change impacts on global rice production. A number of studies have simulated declining rice production due to temperature increase associated with climate change^1^,^2^,^3^. Such yield losses are primarily attributed to changes in phenology. In particular, rising mean daily temperatures from emergence to maturity, i.e. growing season temperature (GST), shortens the vegetative growth^4^ and grain-filling^3^ stages and abridges the duration of vital physiological processes, such as light interception and photosynthesis^5^. Consequently, such processes significantly contribute to simulated yield declines in climate change-crop model studies^6^.

Two recent rice model inter-comparison studies^7^,^8^ showed that rice simulation models predicted both prolonged and shortened growth duration when models were applied to 3–6 °C warmer climate. Such contrasting results indicate uncertainty between model projections and fail to inform what models to use for climate impact studies. Although some degrees of model bias are always occurred, meaningful model prediction requires that model biases do not significantly depart or amplify when applied to warmer climates. Otherwise, unknown bias of model prediction might result in unreliable phenology and yield model projections under warmer climates.

However, maintaining the accuracy of rice phenology simulation has been proven a challenging task^9^. For example, using the ORYZA2000 model^10^, Zhang *et al*.^11^ found that with increasing GST, the simulated growth duration for one rice cultivar gradually departed from the observed despite a satisfactory calibration with the first three years of a 21-year record. By extending the study^11^ and including more observations, van Oort *et al*.^12^ were able to reproduce observed flowering dates, while the bias still increased progressively for the simulated maturity date, despite using all data for model calibration that requires further validation using independent dataset.

To our knowledge, there is no comprehensive assessment of the simulated bias in rice phenology models applied to warmer climates. Modelling experiments often assume no change in cultivar under future climate scenarios^1^,^2^,^3^, as the parameterization is considered cultivar-specific^13^ in most rice simulation models, e.g. ORYZA2000^10^. Excluding the potential influence of cultivar on the phenology simulation bias requires a substantial number of field observations for each cultivar under a wide range of GSTs to enable the resulting difference between the calibration with lower GST and validation with higher GST indicate the accuracy of the phenology model.

The objectives of this study were to (i) evaluate the bias of four phenology models with increasing GST; (ii) quantify the influence of phenology bias on yield simulations; and (iii) identify possible explanations for model bias.

## Results

### Model calibration in lower GST

**Figure 1:**
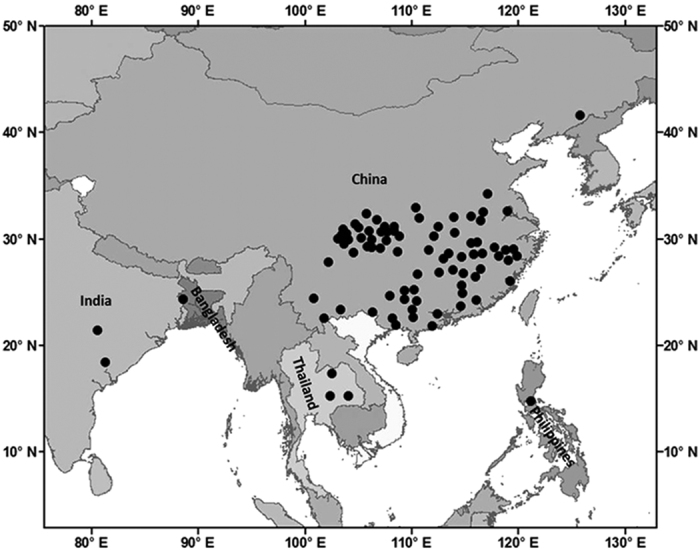
Locations of rice trial sites included in the study. The map was generated with licensed ArcGIS 10.2 using the map of Asia.

To cover the 2–7 °C temperature changes projected by 2100s in the major global rice-growing regions^14^, we collected data for 19 rice cultivars from 775 trials in 86 sites in 5 Asian countries for the period 1981–2009 (Fig. 1), where each cultivar includes between 14 and 260 trials that cover a GST range of 2.2–8.2 °C (Table 1). Here, a combination of cultivar, site and year is referred to as a single trial.

**Figure 2:**
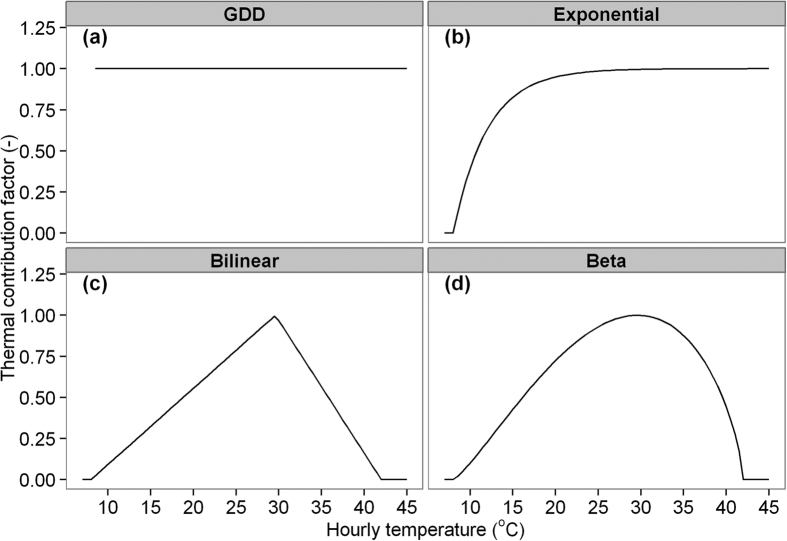
Schematics of the four temperature response functions in the phenology models. (**a**) GDD (*T*_*b*_ = 8); (**b**) exponential (*T*_*b*_ = 8, *TSEN* = 0.25); (**c**) bilinear (*T*_*b*_ = 8, *T*_*o*_ = 30, *T*_*c*_ = 42); and (**d**) beta function (*T*_*b*_ = 8, *T*_*o*_ = 30, *T*_*c*_ = 42; *TSEN* = 1.25).

Four phenology models were examined: growing-degree-day (GDD), exponential, bilinear and beta models (Fig. 2). Description and function of each model can be found in Supplementary Note S1. In short, the four phenology models represent (i) two phenological patterns where the GDD and exponential models assume there is no optimum temperature for phenology development while the bilinear and beta models assume that phenology development firstly accelerates, then decelerates after temperature has exceeded the optimum temperature; and (ii) two modelling curves for phenology, where the GDD and bilinear models use a linear response and the exponential and beta models a non-linear response.

The phenology observations were grouped by the 19 cultivars. We identified the trial with the lowest GST for each cultivar, the GST was then subtracted from the GST for each remaining trial of that cultivar. The difference is referred to as ΔGST. Then we divided the trials for each cultivar group into three temperature intervals for calibration: ΔGST ≤ 1 °C, ≤2 °C, ≤3 °C (here referred to as calibration experiments 1, 2 and 3, respectively), where the trials with lower GST were used for calibration and the remaining for validation (see Supplementary Note S2).

**Figure 3:**
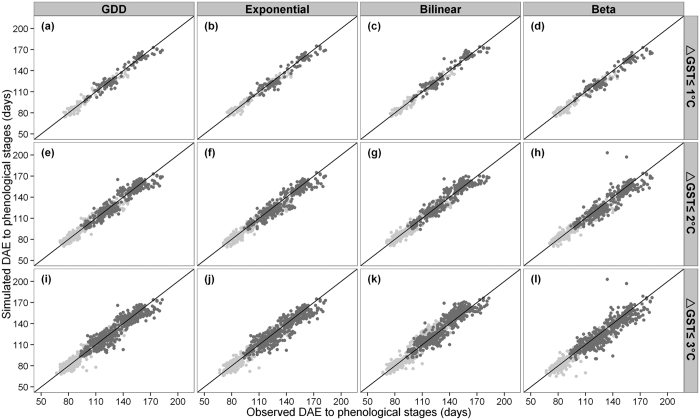
Observed and simulated DAE to flowering (light grey) and DAE to maturity (dark grey) based on the four phenology models (upper x-axis) and three calibration experiments (right y-axis). The solid line is 1:1.

Each phenology model was parameterized for each of the 19 cultivar groups, using an auto-calibration program (see Supplementary Note S3). The graphical comparison (Fig. 3) and linear regression parameters (Table 2) for simulated and observed days after emergence (DAE) to flowering and to maturity are close to 1:1, indicating a good agreement between simulated and observed phenology across all models. The mean absolute deviation (MAD) between the simulated and observed values of DAE to flowering and maturity ranges from 3.5 to 7.2 days, with a normalized root mean square error (NRMSE) between 4.3 and 7.4% (Table 2).

### Model validation in warmer GST

**Figure 4:**
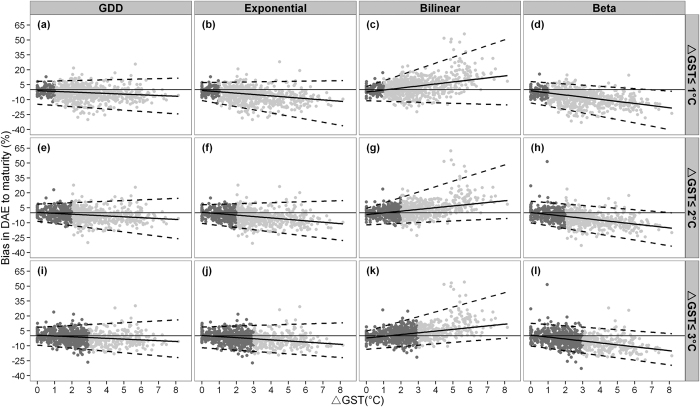
Bias between simulated and observed DAE to maturity expressed per ΔGST for each of the four phenology models (upper x-axis) and three calibration experiments (right y-axis). Solid lines represent the 0.5 percentile regression, lower and upper dashed lines represent the 0.025 and 0.975 percentile regression lines. Dark grey dots illustrate trial data used for calibration and light grey dots data used for validation.

When the phenology models were validated with the higher GST data, their capacity to maintain the same accuracy as the calibration datasets differed. Figure 4 shows the percent bias in DAE to maturity between observed and simulated values. Three regression lines of the percent biases against increasing ∆GST, at three quantiles, 0.025, 0.5 and 0.975, were derived to quantify the lower, middle and upper bias levels (see Supplementary Note S4). Our results show an increase in model biases for DAE to maturity simulation when the bilinear and beta models were applied in warmer GSTs. Specifically, the bilinear model predicted a longer DAE to maturity than that for the observations, as indicated by the augmented upper bias from 5 to 40% and lower bias from −10 to 0% (Fig. 4c,g,k). In contrast, the beta model simulated shorter DAE to maturity with the upper bias decreasing from 5 to 0% and the lower from −10 to −35% (Fig. 4d,h,l). Both the GDD (Fig. 4a,e,i) and exponential (Fig. 4b,f,j) models retained relatively constant bias. Similar results were found when the analysis was repeated for DAE to flowering (see Supplementary Fig. S1).

**Figure 5:**
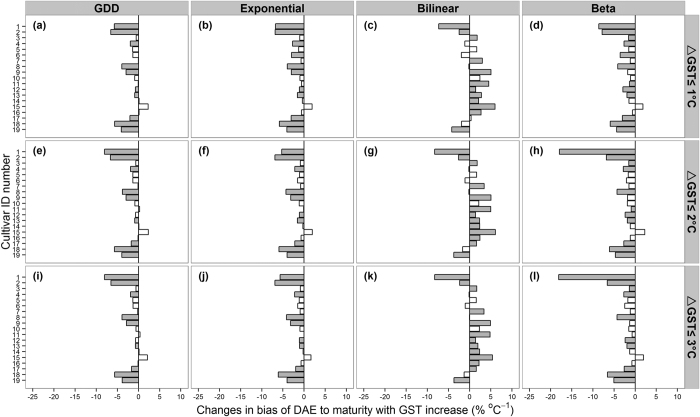
Changes in bias of DAE to maturity with increasing GST per cultivar for each of the four phenology models (upper x-axis) and three calibration experiments (right y-axis). The cultivar ID numbers are presented in Table 1. Grey bars indicate statistically significant bias in yield simulation with GST increase (P < 0.05), and white bars indicate insignificant bias (P > 0.05).

Figure 5 illustrates bias per cultivar for the four phenology models under the three calibration experiments. The model performance varied with cultivar, where the overall most reliable results were derived with the ∆GST ≤ 2 °C calibration dataset using the GDD model for all simulations. For DAE to maturity, the bias was essentially zero for 10 of the 19 cultivars (P > 0.05), whereas the remaining 9 cultivars maintained significant bias (P < 0.05) (Fig. 5e). Similar results for DAE to flowering are presented in Supplementary Fig. S2.

### Influence of phenology bias on yield simulation

**Figure 6:**
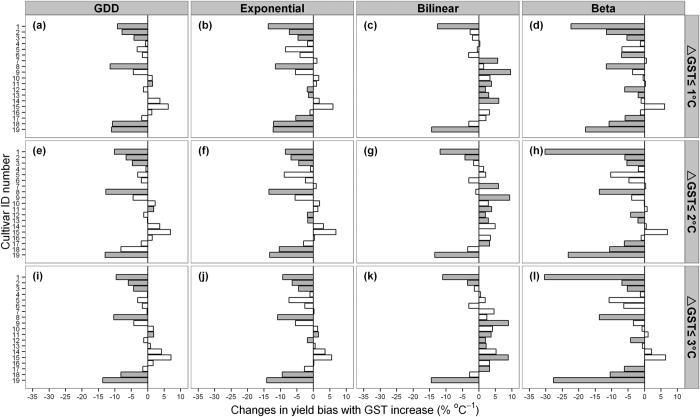
Changes in yield bias with increasing GST per cultivar for each of the four phenology models (upper x-axis) and three calibration experiments (right y-axis). The cultivar ID numbers are presented in Table 1. Grey bars indicate statistically significant bias in yield simulation with GST increase (P < 0.05), and white bars indicate insignificant bias (P > 0.05).

**Figure 7:**
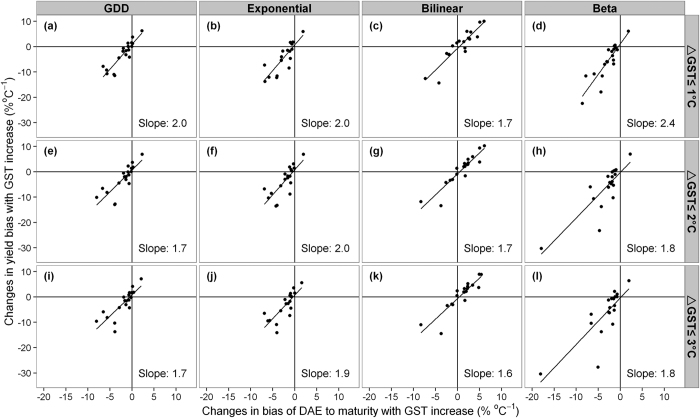
Relation between yield bias and DAE to maturity bias with increasing GST for each of the four phenology models (upper x-axis) and three calibration experiments (right y-axis). Each dot represents a cultivar and the line indicates the ratio of yield bias to phenology bias over all 19 cultivars.

To demonstrate to what extent biased phenology would influence yield simulations under warmer climate, we used the default crop parameters in ORYZA2000 except for those related to phenology, and computed percent bias between simulated yields based on the observed and simulated phenology (see Supplementary Note S4). The model bias for each cultivar under the three calibration experiments is shown in Fig. 6. The direction of the simulated yield bias is consistent with the model performance for DAE to maturity (Fig. 5). The yield bias varied between −30–10% depending on model and calibration experiments (Fig. 6). Notably, on average 1% bias in DAE to maturity in warmer GSTs returned a 2% (1.6–2.4%) yield bias (Fig. 7).

## Discussion

With the unique phenology dataset of 19 cultivars covering a span of 2–8 °C GST, we investigated the performance of four phenology models in cooler and warmer climatic conditions. The calibration results in the lower GST range showed MAD between the simulated and observed DAE to flowering and maturity (3.5–7.2 days with NRMSE between 4.3 and 7.4%) similar to other studies^11^,^15^,^16^,^17^ (2–9 days for MAD and 2–7% for NRMSE). However, under warmer GST, the models’ capacity to maintain accuracy diverted: the GDD and exponential models retained constant bias, while the bilinear model tended to overestimate and the beta model underestimate the duration of growth stages compared to observations. Our study provide further-step results compared with earlier model inter-comparison study^8^ which focused on uncertainty between models but did not demonstrate which models are able to provide better predictability in climate change assessment. Our result indicates that the GDD and exponential models capture the effects of warming better than the bilinear and beta models.

**Figure 8:**
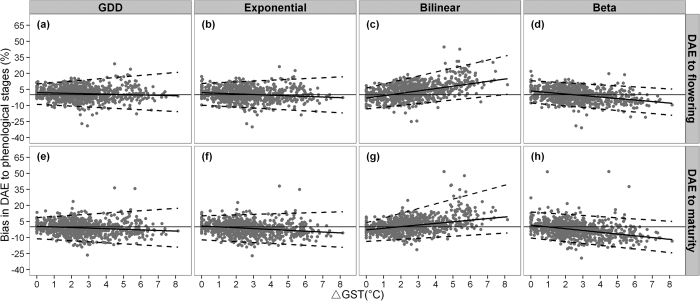
Bias between simulated and observed DAE to flowering and maturity (right y-axis) expressed per ΔGST for each of the four phenology models (upper x-axis) when all data were used for calibration. Solid lines represent the 0.5 percentile regression line, and the lower and upper dashed lines represent the 0.025 and 0.975 percentile regression lines.

Two possible reasons for the gradual increasing bias in the bilinear and beta models are that (i) the models do not reflect actual phenological pattern to climate^11^; and (ii) the size of the calibration data fails to represent the actual phenological pattern and results in incorrect parameterization when there is a significant increase in GST between the calibration and validation datasets^12^. To exclude the latter cause of bias, we used all data as input to the auto-calibration program. The assumption was that a constant model bias across GST for all models would indicate that primarily incorrect parameterization caused the changing predictive biases in Fig. 4. In contrast, if gradually increasing model biases were still present with the bilinear and beta models, this would indicate difficulties in capturing the actual phenology response patterns to climate change of the two models. The resulting bias trends (Fig. 8) were quite similar for all three calibration experiments in Fig. 4. Hence calibrating bilinear and beta phenology models with a range of GST data did not reduce bias under warmer climates, which suggests that the two models are likely to misrepresent phenological patterns under warmer climates.

The bilinear and beta models may be better suited for phenological patterns in chamber experiments^18^, where the phenology development rates accelerated until optimum temperature and then decelerated when the temperature threshold was reached. Similar to our study, recent field-based observation studies found no particular advantage of using the more complex phenology models. For example, the beta model performed better than simpler models for phenology simulation in only one of China’s seven rice-producing regions^7^. Analogous results were found for wheat^6^, where the observed plant development rates in northern India accelerated rather than decelerated when temperatures exceeded optimum temperature. Comparing field and chamber experiments is recommended in further investigations to identify differences in phenological patterns to temperature.

By convention previous climate impact studies^1^,^2^,^3^ have calibrated rice models with only one cultivar. However, our results suggest that such approach may result in biased predictions. Even though the GDD and exponential models give overall consistent phenology responses across GST regimes (Fig. 4), they do not necessarily result in stable predictability for each cultivar (Fig. 5). This indicates that calibration with multiple cultivars and using GDD and exponential models would improve rice phenology predictions.

Another important consequence of phenology bias is the doubling in simulated yield bias (Fig. 6). Similar to studies for rice^8^ and wheat^19^, model uncertainties in simulated yields under optimal water and nutrient conditions, strongly depend on temperature and inherited errors in the phenology simulation. This is because of that phenology determines many basic physiological processes that affect biomass and yield simulation, such as photosynthesis^20^ and light interception^21^. Therefore, our results stress that selecting the model that captures phenology over a range of temperatures is very important for more accurate yield predictions in warmer climates.

Despite the demonstrated advantages of GDD and exponential models for rice phenology and yield simulations, the bilinear model is widely used in rice models^10^,^22^,^23^. These crop models have often simulated that CO_2_-fertilization can offset the negative impacts of increased temperature in rice production in parts of Asia^2^,^24^,^25^, and thus benefit rice production. However, our study showed that the bilinear model frequently overestimated growth duration (Fig. 4), hence the actual yield reduction due to warming may have been underestimated in previous studies, as CO_2_-fertilization may not compensate for the loss. We recommend that the combined effects of CO_2_ and temperature on rice need to be re-evaluated using the GDD or exponential phenology models.

In conclusion, the phenological patterns of bilinear and beta models (where growth decelerates after a certain optimum temperature) resulted in increasing phenology bias under warmer temperatures. The model bias will be doubled when it carry over to yield simulation. More constant phenology biases were achieved with the GDD and exponential models, which simulate phenology without optimum temperature. Moreover, simulations based on multiple cultivars are also able to provide better predictability than using one cultivar. Therefore, for better estimates of climate change-impacts on rice phenology and production, we recommend calibrating with multiple cultivars and using GDD and exponential phenology models.

## Methods

### Dataset

The rice phenology data include field trial observations from agro-meteorological stations in China operated by the China Meteorological Administration, and trials operated by the International Rice Research Institute (IRRI) in the Philippines and its associated institutes in Bangladesh, India and Thailand (Fig. 1). The dataset includes phenology (dates for emergence, transplanting, panicle initiation, flowering and physiological maturity) for 19 cultivars at 86 sites in 775 trials (Table 1), where all except the IR36 cultivar were conducted in China. The dataset includes only cultivars and trials that were carried out under optimal water and nitrogen conditions and with comparable research protocols for phenology measurements for each specific cultivar, to minimize the potential difference in measurement standards between the sites. Daily meteorological data, including sunshine hour, minimum and maximum temperatures, vapour pressure, wind speed and precipitation, were obtained from weather stations at respective site.

### Calibration experiments

Four phenology models were examined: GDD, exponential, bilinear and beta models (algorithms for each model are found in Supplementary Note S1). For the trials sharing one cultivar group, the lowest GST was subtracted from the GST for each trial of the specific cultivar (referred to as ΔGST). For each cultivar group, three calibration experiments were conducted (see Supplementary Note S2). For experiment 1, the calibration data consisted of the trials with ΔGST ≤ 1 °C with the remaining data for validation. For experiments 2 and 3, the calibration data comprised the trials with ΔGST ≤ 2 °C and ≤3 °C. For calibration, we used an auto-calibration program that iterates to return the parameter values with the least difference between observed and simulated phenology for each model (see Supplementary Note S3). For validation, we applied the phenology models and associated calibrated parameters to the trial data with higher GST.

The percent bias between simulated and observed DAE to flowering and maturity was calculated for each cultivar. The changes in model accuracy were measured by regressing percent bias with increasing ∆GST at three quantiles, 0.025, 0.5 and 0.975, to quantify the changes in lower, middle and upper biases as increases in GST (see Supplementary Note S4).

To estimate the error carried over from a biased phenology to yield simulation, we ran ORYZA2000 for each trial with observed phenology dates and phenology simulated using each of the four phenology models. ORYZA2000 was run with potential water and nitrogen conditions with default parameters, except for those related to phenology (default values are available at https://sites.google.com/a/irri.org/oryza2000/downloads). Lastly, we calculated the percent bias between yields generated from observed and simulated phenology (see Supplementary Note S4).

## Additional Information

**How to cite this article**: Zhang, T. *et al*. Model biases in rice phenology under warmer climates. *Sci. Rep.* **6**, 27355; doi: 10.1038/srep27355 (2016).

## Supplementary Material

Supplementary Information

## Figures and Tables

**Table 1:** The genotype, DAE to flowering and maturity coefficient of variation (CV) and the range of GST of 19 cultivars used in the study.

ID number	Cultivars	Genotype	Number of sites (**trials**)	Flowering DAE CV %	Maturity DAE CV %	GST range °C
1	Akihikari	Jap, Inb	1 (21)	4.6	3.7	17.8–20.9
2	Boyou64	Ind, Hyb	6 (22)	5.2	3.8	24.9–28.4
3	Dyou63	Ind, Hyb	14 (33)	10.6	9.0	21.4–26.6
4	Gangyou22	Ind, Hyb	17 (39)	6.6	5.9	21.6–26.5
5	Guangluai4	Ind, Inb	6 (22)	5.0	4.0	22.6–26.2
6	Guangxuan3	Ind, Inb	6 (17)	7.3	6.1	22.9–26.1
7	Guichao13	Ind, Inb	6 (14)	9.4	7.5	21.3–25.4
8	IR36	Ind, Inb	8 (22)	11.8	9.9	25.1–28.8
9	Jinyougui99	Ind, Hyb	11 (25)	6.9	5.0	25.8–29.6
10	Shanyou10	Ind, Hyb	9 (20)	5.7	7.2	25.0–28.2
11	Shanyou2	Ind, Hyb	27 (76)	14.7	11.8	21.2–28.0
12	Shanyou36	Ind, Hyb	6 (18)	21.4	17.1	20.0–28.2
13	Shanyou63	Ind, Hyb	52 (260)	12.8	9.9	21.1–28.6
14	Shanyou64	Ind, Hyb	11 (34)	10.1	8.6	23.6–28.4
15	Shanyougui33	Ind, Hyb	8 (27)	9.9	6.5	24.7–26.9
16	Shanyougui99	Ind, Hyb	9 (28)	9.2	5.8	24.0–28.0
17	Weiyou64	Ind, Hyb	16 (57)	12.1	7.1	22.4–28.0
18	Xieyou64	Ind, Hyb	9 (22)	7.5	6.5	24.0–27.9
19	Zhefu	Ind, Inb	4 (18)	7.3	4.4	21.5–25.2

The cultivars are arranged in alphabetical order. Jap. denotes *Oryza Japonica*; Ind. denotes *Oryza Indica*; Inb. denotes Inbred; Hyb. denotes Hybrid.

**Table 2:** Evaluation of DAE to flowering and maturity for the trials in calibration dataset in the three calibration experiments for the four phenology models.

Phenology Model	DAE	Calibration experiments	Sample size	MAD	*α*	*β*	R^2^	NRMSE
			n trials	days	–	–	–	%
GDD	Flowering	ΔGST ≤ 1 °C	109	3.68	0.96	4.12	0.94	4.55
		ΔGST ≤ 2 °C	289	4.15	0.95	5.12	0.92	5.22
		ΔGST ≤ 3 °C	503	4.68	1.03	−2.33	0.92	5.84
	Maturity	ΔGST ≤ 1 °C	109	4.65	0.94	7.23	0.93	4.33
		ΔGST ≤ 2 °C	289	5.51	0.92	9.37	0.89	5.23
		ΔGST ≤ 3 °C	503	5.95	0.92	9.30	0.85	5.84
Exponential	Flowering	ΔGST ≤ 1 °C	109	3.54	0.95	4.79	0.95	4.29
		ΔGST ≤ 2 °C	289	4.09	0.94	4.65	0.93	5.21
		ΔGST ≤ 3 °C	503	4.48	1.02	−1.77	0.92	5.67
	Maturity	ΔGST ≤ 1 °C	109	4.85	0.93	7.78	0.92	4.46
		ΔGST ≤ 2 °C	289	5.79	0.90	10.92	0.88	5.68
		ΔGST ≤ 3 °C	503	6.25	0.89	11.63	0.84	6.20
Bilinear	Flowering	ΔGST ≤ 1 °C	109	3.54	0.98	1.87	0.95	4.27
		ΔGST ≤ 2 °C	289	4.25	0.99	0.95	0.93	5.16
		ΔGST ≤ 3 °C	503	5.82	1.12	−9.60	0.91	7.38
	Maturity	ΔGST ≤ 1 °C	109	4.41	0.97	4.16	0.93	4.32
		ΔGST ≤ 2 °C	289	5.69	0.96	6.24	0.89	5.31
		ΔGST ≤ 3 °C	503	6.44	0.99	1.86	0.85	6.06
Beta	Flowering	ΔGST ≤ 1 °C	109	3.81	0.92	6.54	0.94	4.68
		ΔGST ≤ 2 °C	289	4.00	0.92	6.90	0.93	5.24
		ΔGST ≤ 3 °C	503	4.23	0.99	0.61	0.91	5.66
	Maturity	ΔGST ≤ 1 °C	109	5.45	0.90	10.43	0.92	5.11
		ΔGST ≤ 2 °C	289	6.53	0.90	11.19	0.82	6.90
		ΔGST ≤ 3 °C	503	7.19	0.91	9.21	0.79	7.38

MAD: mean absolute deviation; *α*: slope of linear regression between simulated and observed values; *β*: intercept of linear regression between simulated and observed values; *R*^*2*^: coefficient of determination; *NRMSE*: normalized root mean square error. The equations are available in Supplementary Note S3.

## References

[b1] Matthews R., Kropff M., Horie T. & Bachelet D. Simulating the impact of climate change on rice production in Asia and evaluating options for adaptation. Agr. Syst. 54, 399–425 (1997).

[b2] Yao F., Xu Y., Lin E., Yokozawa M. & Zhang J. Assessing the impacts of climate change on rice yields in the main rice areas of China. Clim. Chang. 80, 3–4 (2007).

[b3] Aggarwal P. & Mall R. Climate change and rice yields in diverse agro-environments of India. II. Effect of uncertainties in scenarios and crop models on impact assessment. Clim. Chang. 52, 331–343 (2002).

[b4] Zhang T., Huang Y. & Yang X. Climate warming over the past three decades has shortened rice growth duration in China and cultivar shifts have further accelerated the process for late rice. Glob. Change Biol. 19, 563–570 (2013). 10.1111/gcb.12057 23504793

[b5] Wassmann R. . Climatic change affecting rice production: the physiological and agronomic basis for possible adaptation strategies. Adv. Agro. 101, 59–122 (2009).

[b6] Lobell D. B., Sibley A. & Ortiz-Monasterio J. I. Extreme heat effects on wheat senescence in India *Nat*. Clim. Change, 2, 186–189 (2012).

[b7] Zhang S. & Tao F. Modeling the response of rice phenology to climate change and variability in different climatic zones: Comparison of five models. Eur. J. Agron. 45, 165–176 (2013).

[b8] Li T. . Uncertainties in predicting rice yield by current crop models under a wide range of climatic conditions. Glob. Change Biol. 21, 1328–1341 (2015). 10.1111/gcb.12758 25294087

[b9] Meinke H. Agricultural impacts: Europe’s diminishing bread basket. Nat. Clim. Change 3, 541–542 (2014).

[b10] Bouman B. A. M. . ORYZA2000: Modeling Lowland Rice. (eds Bouman B. A. M. .) (Wageningen, The Netherlands, 2001).

[b11] Zhang T., Zhu J. & Yang X. Non-stationary thermal time accumulation reduces the predictability of climate change effects on agriculture. Agric. Forest Meteorol. 148, 1412–1418 (2008).

[b12] Van Oort P. A. J., Zhang T., de Vries M. E., Heinemann A. B. & Meinke H. Correlation between temperature and phenology prediction error in rice (Oryza sativa, L.). Agric. Forest Meteorol. 151, 1545–1555 (2011).

[b13] Li T. . Simulation of genotype performances across a larger number of environments for rice breeding using ORYZA2000. Field Crops Res. 149, 312–321 (2013).

[b14] IPCC. Summary for policymakers. In Climate Change 2014: Impacts, Adaptation, and Vulnerability. Part A: Global and Sectoral Aspects. Contribution of Working Group II to the Fifth Assessment Report of the Intergovernmental Panel on Climate Change (ed. Field C. B. .), 1–12 (Cambridge, 2014).

[b15] Yin X., Kropff M., Horie T., Nakagawa H. & Goudriaan J. A model for photothermal responses of flowering in rice II. Model evaluation. Field Crops Res. 51, 201–211 (1997).

[b16] Gao L., Jin Z., Huang Y. & Zhang L. Rice clock model: a computer model to simulate rice development. Agric. Forest Meteorol. 60, 1–16 (1992).

[b17] Timsina J. & Humphreys E. Performance of CERES-Rice and CERES-Wheat models in rice-wheat systems: A review. Agri. Sys. 90, 1–3 (2006).

[b18] Yin X., Kropff M. & Goudriaan J. Differential effects of day and night temperature on development to flowering in rice. Annals of Botony. 77, 203–213 (1996).

[b19] Asseng S. . Uncertainty in simulating wheat yields under climate change. Nat. Clim. Change. 3, 827–832 (2013).

[b20] Craufurd P. & Wheeler T. Climate change and the flowering time of annual crops. J. Exp. Bot. 60, 2529–2539 (2009). 19505929 10.1093/jxb/erp196

[b21] Mishra A., Singh R., Raghuwanshi N., Chatterjee C. & Forebrich J. Spatial variability of climate change impacts on yield of rice and wheat in the Indian Ganga Basin. Sci Total Environ., 468–469, S132–138 (2013). 10.1016/j.scitotenv.2013.05.080 23800620

[b22] Ritchie J. . Understanding options for agricultural production (eds Ritchie .) (Kluwer, 1996).

[b23] Keating B. . An overview of APSIM, a model designed for farming systems simulation. Eur. J. Agron. 18, 267–288 (2003).

[b24] Lin E. . Climate change impacts on crop yield and quality with CO_2_ fertilization in China. Philos. Trans. R. Soc. B 360, 2149–2154 (2005). 10.1098/rstb.2005.1743 PMC1569568 16433100

[b25] Kim H., Ko J., Kang S. & Tenhunen J. Impacts of climate change on paddy rice yield in a temperate climate. Glob. Change Biol. 19, 548–562 (2013). 10.1111/gcb.12047 23504792

