# Supplementary material for: Model biases in rice phenology under warmer climates
Source: Sci Rep. 2016 Jun 7;6:27355. doi: 10.1038/srep27355 (PMC4895141; doi:10.1038/srep27355)
Supplement: Supplementary Information [file srep27355-s1.doc]

Supplementary online material

for

**Model biases in rice phenology under warmer climates**

Tianyi Zhang^1,6*^, Tao Li^2^, Xiaoguang Yang^3^, Elisabeth Simelton^4,5^

1. State Key Laboratory of Atmospheric Boundary Layer Physics and Atmospheric Chemistry, Institute of Atmospheric Physics, Chinese Academy of Sciences, Beijing, 100029, China; 2. International Rice Research Institute, Los Baños, Philippines; 3. College of Resources and Environmental Sciences, China Agricultural University, Beijing, China; 4. World Agroforestry Centre (ICRAF), Ha Noi, Viet Nam; 5. Sustainability Research Institute, University of Leeds, Leeds, UK

*Corresponding author: Dr. Tianyi Zhang, zhangty@post.iap.ac.cn; Tel: +86 10 62 012 067

Supplementary Notes: S1, S2, S3, and S4

Supplementary Table S1

Supplementary Figures: S1and S2.

References of supplementary

**Note S1. Descriptions of the rice phenology models**

Current rice phenology models typically involve three processes: temperature response function, photoperiod function, and transplanting shock function. Temperature response function is the most important process for rice phenology development^1^. Therefore, we examine the four most common temperature response functions in rice models: growing-degree-day (GDD), exponential, bilinear and beta phenology models (See Fig. 2 in the main text).

In all models the phenological process is simulated based on hourly temperatures ($T_{h}$), calculated from daily minimum ($T_{\min}$) and maximum ($T_{\max}$) temperatures (Eq. 1).

$T_{h}=\frac{T_{\min}+T_{\max}}{2}+(T_{\max}-T_{\min})\frac{\cos(0.2618(h-14))}{2}$ (Eq. 1)

where $h$ is the time of day.

The daily thermal time (*H_d_*, °C d) is the average hourly thermal time above the base temperature (*T_b_*, °C) at which the plant phenology starts, modified by a *TF* at *T_h_* (*TF_h_,* varies between 0 and 1) and the photoperiod effect in the photoperiod-sensitive phase (*EFP*, varies between 0 and 1) (Eq. 2).

$H_{d}=\frac{(\sum_{h=1}^{24} (T_{h}-T_{b})\times TF_{h})\times EFP}{24}$ (Eq. 2)

where *TF_h_* defines the response of phenology development to temperature represented in each of the four phenology models.

(1) The GDD model (Eq. 3) assumes a constant thermal contribution factor when *T_h_* ≥ *T_b_*.

$TF_{h}=\left\{\begin{matrix} 0 & (T_{h}<T_{b}) \\ 1 & (T_{h}\geq T_{b}) \end{matrix}\right.$ (Eq. 3)

(2) The exponential model (Eq. 4) assumes that when *T_h_* ≥ $T_{b}$, the rate of increase in the thermal contribution factor gradually slows down, with the higher temperature indefinitely closing at 1,

$TF_{h}=\left\{\begin{matrix} 0 & (T_{h}<T_{b}) \\ 1-e^{-TSEN(T_{h}-T_{b})} & (T_{h}\geq T_{b}) \end{matrix}\right.$ (Eq. 4)

where, $TSEN$ is the phenological parameter determining the curvature. The phenology development asymptotically increases with increasing temperature. The *TF_h_* of the exponential model changes slower compared to GDD per unit temperature increase.

(3) The bilinear model (Eq. 5) demonstrates a process in which $TF_{h}$ increases linearly from $T_{b}$ to the optimum temperature ($T_{o}$) for phenology development; thereafter, it decreases linearly until it reaches the ceiling temperature ($T_{c}$), at which point the phenology development stops.

$TF_{h}= \left\{\begin{matrix} 0 & (T_{h}<T_{b} or T_{h}>T_{c}) \\ (T_{h}-T_{b})/(T_{o}-T_{b}) & (T_{b}\leq T_{h}<T_{o}) \\ (T_{c}-T_{h})/(T_{c}-T_{o}) & (T_{o}\leq T_{h}\leq T_{c}) \end{matrix}\right.$ (Eq. 5)

(4) The beta model (Eq. 6) has a similar temperature response curve to the bilinear model, but is expressed in a non-linear manner with a slower change of *TF_h_* per degree temperature increase.

$TF_{h}=\left\{ \begin{matrix} 0 & (T_{h}<T_{b} or T_{h}>T_{c}) \\ \left[\left(\frac{T_{h}-T_{b}}{T_{o}-T_{b}}\right)\left(\frac{T_{c}-T_{h}}{T_{c}-T_{o}}\right)^{\left(T_{c}-T_{o}/T_{o}-T_{b}\right)}\right]^{TSEN} & (T_{b}\leq T_{h}\leq T_{c}) \end{matrix}\right.$ (Eq. 6)

For the functions simulating photoperiod (Eq. 7) and transplanting shock effects (Eq. 8), we adopted the algorithms in ORYZA2000.

$EFP=\left\{\begin{matrix} 1 & DL<DL_{m}, or DVS\notin PPD_{sen} \\ \min\{1,\max[0,1-P_{s}(DL-DL_{m})]\} & DL\geq DL_{m}, and DVS\in PPD_{sen} \end{matrix}\right.$ (Eq. 7)

where *EFP* is the effect of the day length on *H_d_* in the photoperiod-sensitive (*PPD_sen_*) phase of the development stage (DVS); *DL* is the day length; *DL_m_* is the maximum optimum photoperiod, and *P_s_* is a photoperiod sensitivity parameter.

*TSHCKD* denotes that the thermal time accumulation is delayed before rice resumes growth (Eq. 8) and is only activated after transplanting from the seedbed

$TSHCKD=SHCKD\times TSTR$ (Eq. 8)

where *SHCKD* is the transplanting shock parameter, and $TSTR$ the sum of $H_{d}$ from emergence to transplanting.

**Note S2. Calibration and validation datasets and experiments**

To estimate the potential model bias under warmer climates, we calibrated the four phenology models with the trial data having lower growing season temperature (GST) and validated with trials having a higher GST. For each cultivar the trial with the lowest GST was subtracted from the GST of each trial for the same cultivar. The difference is referred to as $\Delta\text{GST}$ (Eq. 9).

$\Delta GST_{c,i}=GST_{c,i}-LowestGST_{c}$ (Eq. 9)

where $GST_{c,i}$ is the GST in trial *i* for cultivar *c*; $LowestGST_{c}$ is the lowest GST of the group of trials for cultivar *c*, and $\Delta GST_{c,i}$is the GST difference of trial *i* relative to $LowestGST_{c}$.

Using three $\Delta\text{GST}$ thresholds, we composed three calibration experiments for each cultivar. The calibration datasets included:

(1)$\Delta\text{GST}\leq1^{\text{o}}\text{C}$: trials with no higher than 1°C $\Delta\text{GST}$ for each cultivar;

(2)$\Delta\text{GST}\leq2^{\text{o}}\text{C}$: trials with no higher than 2°C $\Delta\text{GST}$ for each cultivar;

(3)$\Delta\text{GST}\leq3^{\text{o}}\text{C}$: trials with no higher than 3°C $\Delta\text{GST}$ for each cultivar.

The remaining trial data in each experiment was used for model validation.

**Note S3. Model calibration**

To avoid manual calibration, we employed an auto-calculation program. Referring to Soundharajan and Sudheer (2013)^2^, our auto-calculation program was based on differential evolution algorithms of global optimization^3^ and is available online for download: (https://sites.google.com/a/irri.org/oryza2000/downloads/new-release/download-new-version). The program optimizes model parameters by minimizing the errors between simulated and observed phenology through the iteration of different combinations of model parameters. The program is set with a theoretical value range for each parameter to retrieve the best-fit values within the range. Here, the parameter ranges were taken from the literature (Table S1) to ensure biophysically meaningful values for rice.

Table S1. Parameter range for iteration in auto-calibration

| **Parameters** | **Ranges** | **References** |
| --- | --- | --- |
| ***Temperature response*** ^Note1^ |  |  |
| $T_{b}$ | 6.0–13.0 | Bouman et al^5^  Ebata^6^  Gao et al^7^  Matthews et al^8^ |
| $T_{o}$ | 27.3–31.9 | Yin et al^9^ |
| $T_{c}$ | 40.0–43.0 | Bouman et al^5^  Ebata^6^  Gao et al^7^  Matthews et al^8^ |
| $TSEN$ (exponential model) | 0.01–1.0 | See Note 2 |
| $TSEN$ (beta model) | 0.0–2.5 | See Note 2 |
| ***Photoperiod effect*** ^Note1^ |  |  |
| *DL_m_* | 11.3–12.5 | Matthews et al^8^ |
| *P_s_* | 0.0–0.35 | Matthews et al^8^ |
| ***Transplanting shock effect*** ^Note3^ |  |  |
| $SHCKD$ | 0.2–0.6 | Bouman et al^5^ |

Note 1: The parameter range for the temperature response curve was based on studies by Bouman et al.^5^ , Ebata^6^, Yin et al.^7^, and Matthews et al.^8^. The range for $T_{o}$ was derived from chamber experiments made by Yin et al.^7^ , however, four cultivars in that study (i.e., IR42, CO36, MR84, PETA) were disregarded due to poor fit (more than one standard deviation). The parameter ranges for the photoperiod effect were based on trials in the study by Matthews et al.^8^.

Note 2: *TSEN* in exponential and beta models is an empirical parameter to fit model rather than a physiological parameter. We set the range by a trial-and-error method; i.e. enable auto-calibration program retrieve model parameters within an accepted time cost.

Note 3: Relevant experimental data on the transplanting shock effect is limited; therefore, we assumed a 50% range with the default value (0.4) in ORYZA2000.

Five parameters for goodness-of-fit were used to quantify the modeling performance of the calibration dataset: mean absolute deviation (*MAD*, Eq. 10), slope (*α*, Eq. 11) and intercept (*β*, Eq. 11) of linear regression between simulated and observed values, coefficient of determination (*R^2^*, Eq. 12), and normalized root mean square error (*NRMSE*, Eq. 13), where *sim* denotes the simulated values, *obs* the observed values in the trial *i* and $\overline{obs}$ the mean of all observations; and *n* is the number of data.

$MAD=\frac{\sum|sim_{i}-obs_{i}|}{n}$ (Eq. 10)

$sim_{i}=\beta+\alpha obs_{i}+\varepsilon$ (Eq. 11)

$R^{2}=\left(\frac{n\sum sim_{i}obs_{i}-\sum sim_{i}\sum obs_{i}}{\sqrt{n\sum sim_{i}^{2}-(\sum sim_{i})^{2}}\sqrt{n\sum obs_{i}^{2}-(\sum obs_{i})^{2}}}\right)^{2}$ (Eq. 12)

$NRMSE=\frac{\sqrt{\sum(sim_{i}-obs_{i})^{2}/n}}{\overline{obs}}100\%$ (Eq. 13)

**Note S4. Model validation**

To validate the model performance under warmer climates, we applied the parameterization values for the calibrated models to the trial data with higher GST. We calculated the percent bias between simulated and observed number of days after emergence (DAE) to flowering or to maturity for each cultivar (Eq. 14)

$DAE_{bias}=\frac{DAE_{sim}-DAE_{obs}}{DAE_{obs}}\times100\%$ (Eq. 14)

where *DAE_bias_* represents the percent bias between the simulated (*DAE_sim_*) and observed DAE (*DAE_obs_*) to flowering and maturity.

To estimate the bias with increasing ∆GST we used quantile regression^4^  at three quantiles 0.025, 0.5 and 0.975, to quantify the changes in lower, middle and upper biases. The three lines along the x-axis indicate the changes in model accuracy with increasing GST.

To estimate the error carried over from a biased phenology to yield simulation, we ran ORYZA2000 for each trial with (1) observed phenology and (2) phenology simulated by each of the four phenology models. ORYZA2000 was run with potential water and nitrogen conditions and default parameters, except for those related to phenology (default values for ORYZA2000 can be downloaded from https://sites.google.com/a/irri.org/oryza2000/downloads). The percent bias between yields simulated from observed and modeled phenology $Yield_{bias}$ (Eq. 15), is calculated as

$Yield_{bias}=\frac{Yield_{simPhenology}-Yield_{obsPhenology}}{Yield_{obsPhenology}}\times100\%$ (Eq. 15)

where, $Yield_{simPhenology}$ is the simulated yield based on phenology outputs from the four calibration experiments, and $Yield_{obsPhenology}$ the simulated yield from based on observed phenology. Eq. 15 presents the relative error in yield simulation resulting from the biased phenology simulation.


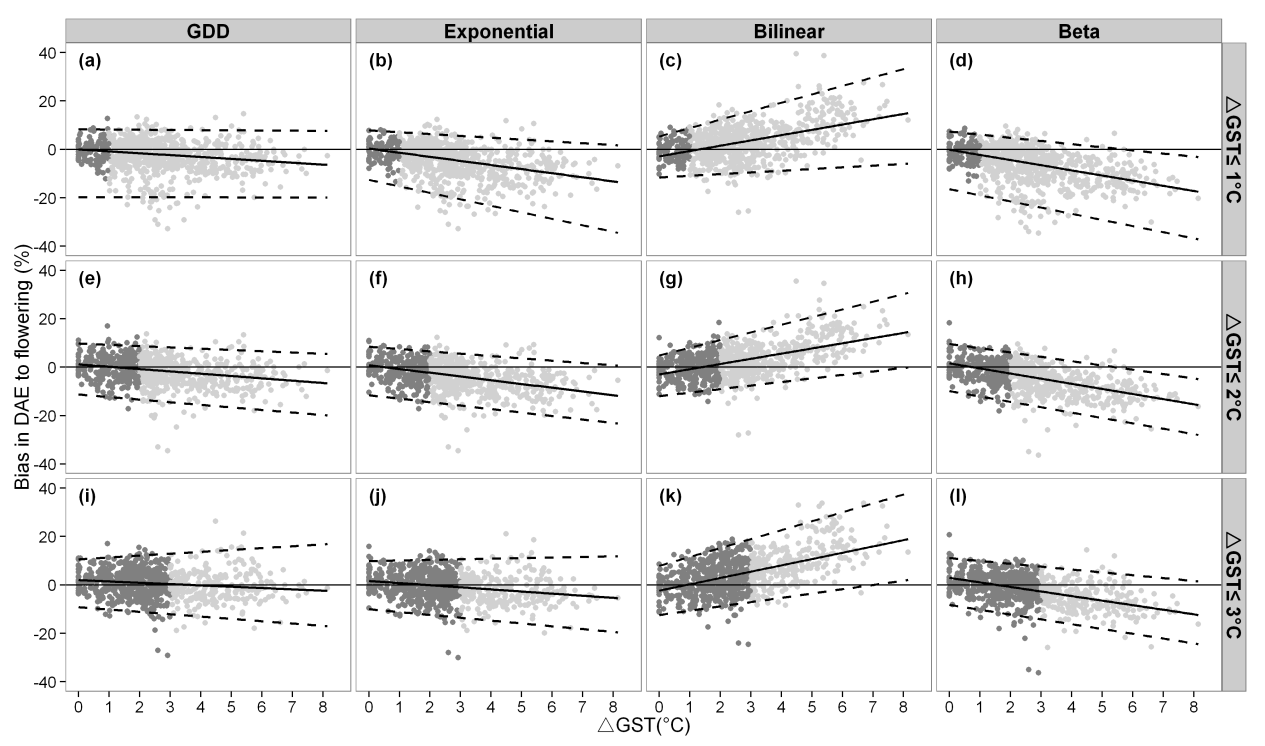


Fig. S1. Bias between simulated and observed DAE to flowering expressed per $\Delta\text{GST}$ for each of the four phenology models (upper x-axis) and the three calibration experiments (right y-axis). Solid line represents the 0.5 percentile regression line, the lower and upper dashed lines represent the 0.025 and 0.975 percentile regression lines. Dark grey dots illustrate trial data used for calibration, and light grey dots data for validation.


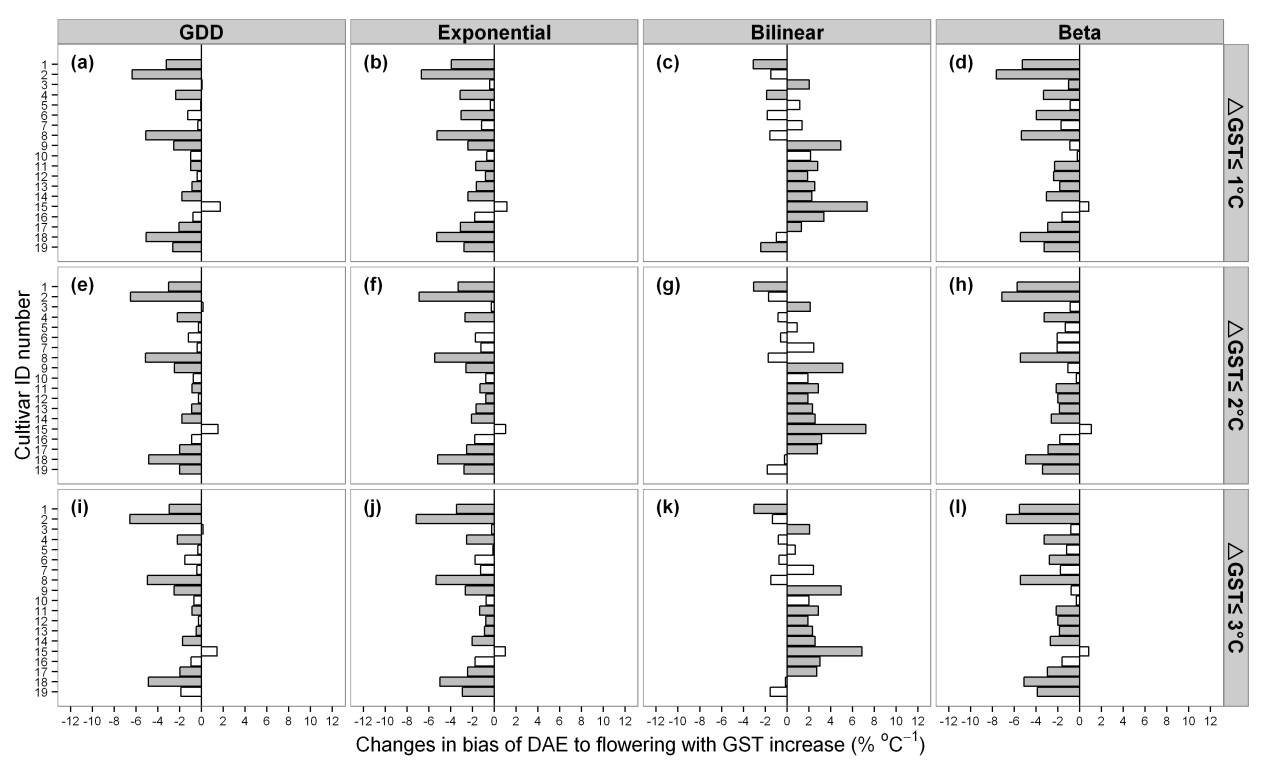


Fig. S2. Changes in DAE to flowering with increasing GST per cultivar for each of the four phenology models (upper x-axis) and the three calibration experiments (right y-axis). The cultivar ID numbers are presented in Table 1 in the main text. Grey bars indicate statistically significant bias in yield simulation with GST increase (P<0.05), and white bars indicate insignificant bias (P>0.05).

**References**

1. Ceglar, A., Crepinsek, Z., Kajfez-Bogataj, L. & Pogacar, T. The simulation of phenological development in dynamic crop model: The Bayesian comparison of different methods. *Agric. Forest Meteorol.* **151**, 101–115 (2011).
2. Soundharajan, B. & Sudheer, K. Sensitivity analysis and auto-calibration of ORYZA2000 using simulation-optimization framework. *Paddy Water Environ.* **11**, 1 – 4 (2013).
3. Storn, R. & Price, K. Differential evolution - a simple and efficient adaptive scheme for global optimization over continuous spaces: Technical Report International Computer Science Institute, Berkley (1995).
4. Koenker, R. & Bassett, G. Jr. Regression Quantiles. *Econometrica* **46**, 33 – 50 (1978).
5. Bouman, B. A. M. *et al.* *ORYZA2000: Modeling Lowland Rice.* (eds Bouman, B.A.M. *et al.*) (Wageningen, The Netherlands, 2001)
6. Ebata, M. Effective heat unit summation and base temperature on the development of rice plant. *Japan. Jour. Crop Sci*. **59**, 225–232 (1990).
7. Gao, L., Jin, Z., Huang, Y. & Zhang, L. Rice clock model: a computer model to simulate rice development. *Agric. Forest Meteorol.* **60**, 1–16 (1992).
8. Matthews, R., Kropff, M., Bachelet, D., van Laar, H. H. *Modeling the impact of climate change on rice production in Asia* (eds. Matthews *et al.*) (1995)
9. Yin, X., Kropff, M. & Goudriaan, J. Differential effects of day and night temperature on development to flowering in rice. *Annals of Botony.* **77**, 203 – 213 (1996).
